# Insulin reverses choriocarcinoma 5- fluorouracil resistance

**DOI:** 10.1080/21655979.2021.1931643

**Published:** 2021-05-26

**Authors:** Ying Shan, Yanyi Li, Hongyu Han, Cui Jiang, Hu Zhang, Jiachang Hu, Huanmei Sun, Jianglong Zhu

**Affiliations:** a Department of Obstetrics and Gynecology, Shanghai Pudong Hospital, Fudan University Pudong Medical Center, Pudong, China; b Department of Health Science, Graduate School of Medical, Osaka University, Osaka, Japan

**Keywords:** Choriocarcinoma, chemoresistance, insulin, 5-FU

## Abstract

Choriocarcinoma (CC) is a gestational trophoblastic tumor secondary to a gravid or non-gravid pregnancy. It is characterized by rapid growth, high invasion, and high metastatic potential and chemotherapy resistance that significantly affect survival rate of CC patients. Insulin is implicated in alleviation of chemotherapy resistance in CC. However, the mechanism of reversing resistance in CC has not been explored. Our purpose was to explore insulin effect on 5-fluorouracil (5-FU) resistance in CC and elucidate its potential mechanism *in vitro* and *in vivo*. CKK-8, colony formation, Transwell, and flow cytometry were used to detect the effect of insulin on 5-FU resistance in CC cells JEG-3 and JARS. Xenograft mice were used to evaluate the effect of insulin on 5-FU resistance. Results showed that insulin combined with 5-FU suppressed cell viability by 30% in JEG-3 and 43% in JAR compared with 5-FU alone in 72 h. What’s more, insulin combined with 5-FU promoted cell apoptosis, inhibited cell proliferation, migration, and phosphorylation of survivin at residue threonine 34 (Thr34) and drug resistance-related proteins, P-GP and MRP1 levels (*p* < 0.05). *In vivo* experiment showed Insulin combined with 5-FU suppressed tumor volume by 35% compared with 5-FU alone and 73% compared with control in CC xenograft mice. In summary, the findings of this study show that insulin reversed chemoresistance of CC cells to 5-FU by inhibiting phosphorylation of survivin. Development of a therapeutic strategy that combines insulin with the chemotherapeutic agent 5-FU has a great potential in improving survival of CC patients.

## Graphical abstract

**Figure:**
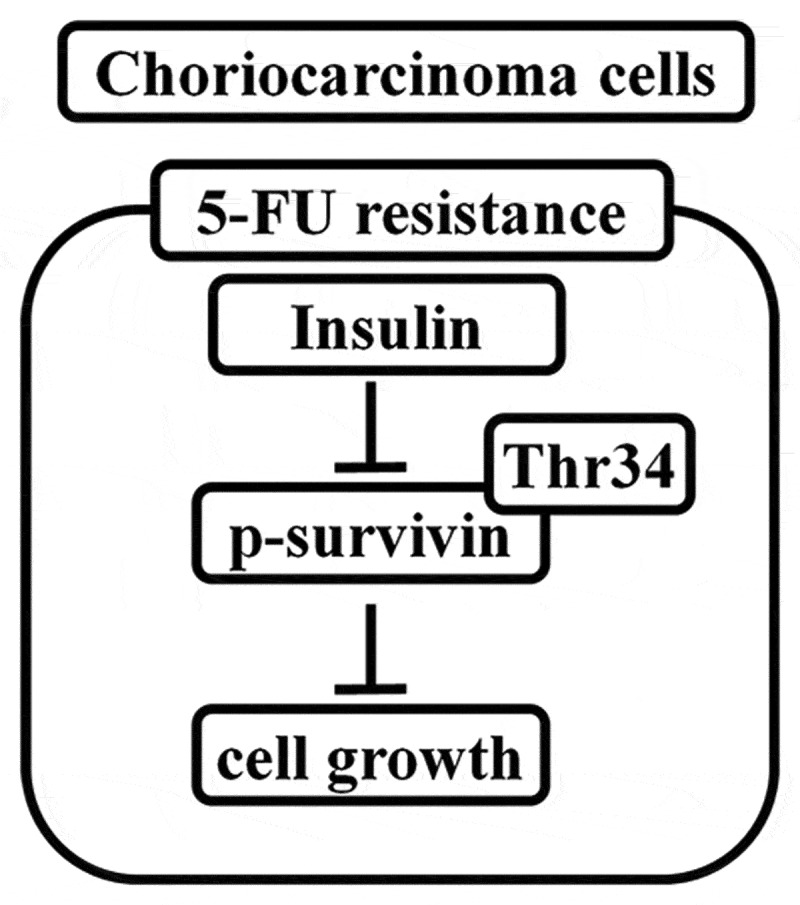

## Introduction

Choriocarcinoma (CC) is a malignant trophoblastic tumor. It is associated with hypodifferentiated somatic cell carcinoma as a component of germ cell tumors [1]. CC mainly results from excessive invasion of the endometrium by trophoblastic cells, making CC highly aggressive [2]. CC occurs both in gestational and non-gestational periods, but occurs predominantly during pregnancy. Notably, more than 50% of CCs in pregnancy develop from complete chylothorax (CHM). In addition, miscarriage, normal full-term or preterm delivery, and ectopic pregnancy may result into CC [3–5]. Previous studies report that CC is characterized by distant metastasis to the lungs, brain, vagina, liver, kidneys, and other organs due to its high invasiveness and blood dissemination [6–8]. Previous treatment approaches for CC mainly included supportive treatment such as chemotherapy, surgery, radiotherapy, and interventional procedures [9]. Although these treatment methods reduced mortality rate of CC from 90% to 20%, complete treatment is not achieved in 10% of patients with stages 2 and 3 as based on the International Society of Gynecology and Obstetrics (FIGO) classification [10]. This limitation is attributed to chemotherapy drug resistance and death of patients with these stages due to high recurrence and metastasis rate [11]. 5-Fluorouracil (5-FU) is a cell cycle-specific chemotherapeutic drug that targets the S-phase tumor cells. It is effective in the treatment of CC; however, it has severe toxic effects that reduce lifetime quality and survival rate of patients [12,13]. Therefore, studies should explore a chemotherapeutic potentiator to improve the effect of chemotherapeutic drugs and reduce the toxic side effects on patients. Cytotoxic effect of chemotherapy as the main treatment method for malignant tumors is limited by lack of selectivity, which leads to damage of normal tissue cells. Therefore, there is need to develop methods to improve sensitivity of tumors to chemotherapeutic drugs and reduce toxic side effects of chemotherapeutic drugs.

Insulin is a protein hormone, which is secreted by pancreatic β-cells in the pancreas under stimulation of various internal and external substances. It is the only glucose-lowering hormone in the body; therefore, it can promote protein, fat, and glycogen synthesis [14,15]. Recent studies have extensively explored the mechanism of insulin in promoting tissue cell metabolism level. Insulin is a hormone with multiple biological effects and is the main hormone-regulating metabolism in the body. Insulin enhances metabolic level and promotes synthesis of RNA and DNA. In addition, it acts as a potentiator to promote synthesis of genetic material and stimulate the growth and proliferation of normal cells and tumor cells. Furthermore, it induces tumor cells to enter the proliferation cycle, thus improving sensitivity of tumor cells to chemotherapeutic drugs [16]. Besides, insulin enhances cisplatin-induced apoptosis in human esophageal squamous cell carcinoma EC9706 cells [17]. Insulin enhances the effects of chemotherapeutic agents on colorectal cancer while single using not showing toxicity [18].

Since the role of insulin CC remains unknown, our purpose was to assess the effect of insulin and explore its mechanism of action on CC resistant to 5-FU.

## Methods

### Cell culture and transfection

Human CC cells (JEG-3 and insulin cells) were purchased from the Cell Bank of the Chinese Academy of Sciences (Shanghai, China). Cells were cultured in 90% Dulbecco modified Eagle’s medium (DMEM; Gibco, Grand Island, NY) supplemented with 10% fetal bovine serum (Gibco), 100 U/mL penicillin, and 100 μg/mL streptomycin in a 37°C humidified incubator with 5% CO_2_. Cells were transfected with Lipofectamine 2000 (Invitrogen) following the manufacturer’s protocol.

## Cell viability assay

Cell Counting Kit-8 (CCK-8) assay was used to detect cell survival rates. Transfected cells were seeded into 96-well plates at a density of 5,000 cells/well in triplicate. Cell viability was measured with CCK-8 system (Gibco) at different cisplatin concentrations after seeding, according to the manufacturer’s instructions.

For colony formation assays, transfected cells were seeded into six-well plates at a density of 2,000 cells/well and maintained in DMEM media containing 10% fetal bovine serum for 10 days. Colonies were observed under a microscope and counted after they were fixed and stained.

## Xenograft tumor formation in mice

JAR cells (3 × 10^6^) were counted and resuspended with 100 µL RPMI 1640 and injected subcutaneously into BALB/c female nude mice (purchased from the animal department of Fudan University with approval of the Animal Care and Use Committee of Fudan University), and then tumor sizes were measured at the indicated time. After determination of tumor sizes, 20 mg/kg i.p. of 5-FU, 2.5 U/kg i.p. of insulin were administered daily three times a week for 5 weeks. Tumor volume was calculated as tumor volume = length × width^2^. Tumors were excised 5 weeks after administration of the different treatments.

## Statistical analysis

All results in this study were statistically analyzed using Prism 7.0 GraphPad Prism 7.0 (GraphPad Software, La Jolla, CA, USA). *T*-test was performed to explore differences of the two independent groups. One-way analysis of variance test was applied for the determination of differences among various groups. Kaplan–Meier curves and the log-rank test were used to analyze survival rate of patients. *p* < 0.05 was considered statistically significant.

More detailed materials and methods are presented in the Supplementary Methods.

## Results

Here, we aim to verify the effect of insulin and potential role on CC resistant to 5-FU and found that insulin combined with 5-FU suppressed CC tumor progression via inhibiting the phosphorylation of survivin. Therefore, our data for the first investigated the functional of insulin in CC, providing new insights into the pathogenesis of CC chemoresistance.

## Insulin sensitizes CC cells to 5-FU *in vitro*

**Figure 1.**
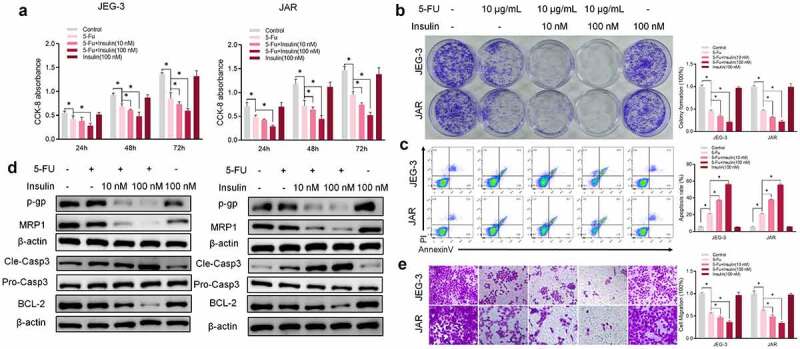
Insulin sensitizes choriocarcinoma cells to 5-FU *in vitro*. (a) CCK-8 assays were used to evaluate cell proliferation at different times. (b) Colony formation assay showing proliferation of JEG-3 and JARS cells cultured in 5-FU or insulin for 48 h. (c) Quantification and analysis of apoptosis rates of JEG-3 and JARS cells cultured in 5-FU or insulin for 48 h using flow cytometry. (d) Western blot analysis showing protein expression levels of p-gp, MRP1, cleaved caspase-3 (Cle-Casp3), pro caspase-3 (Pro-Casp3), and BCL-2 in JEG-3 and JARS cells. β-Actin was used as the internal reference. (e) Cells migration rate detection using Transwell assay. Data are expressed as mean ± SEM (*n* = 3; *p < 0.05)

To assess the role of insulin in 5-FU resistance *in vitro*, two CC cells line JEG-3 and JAR were used to test insulin effect on cell viability. Analysis of CCK-8 results showed that a single dose of insulin had no effect on CC cells proliferation compared with the control group (Figure 1a). On the contrary, co-treatment with insulin (10 nM and 100 nM) and 5-FU significantly enhanced sensitivity of JEG-3 and JAR to 5-FU. Similar results were obtained from colony formation analysis (Figure 1b). To explore the effect of insulin on apoptosis, cells were stained with Annexin V and analyzed using flow cytometry. Insulin co-administered with 5-FU significantly promoted apoptosis in CC cells compared with administration of 5-FU alone (Figure 1c). Similarly, western blot analysis of apoptosis markers (caspase-3 and BCL-2) showed significantly high pro-apoptotic effects of insulin combined with 5-FU. Moreover, insulin combined with 5-FU suppressed drug resistance-related proteins P-GP and MRP1 (Figure 1d). However, single dose of insulin had no significant effect on the activity of 5-FU against cancer cells. Transwell assays were used to assess the effect of insulin on cell migration. Analysis showed that migration of CC cells was significantly inhibited after treatment with both insulin and 5-FU (Figure 1e). These findings show that co-administration of insulin with 5-FU significantly affects CC cell functions such as apoptosis, proliferation, metastasis, and drug resistance, and insulin increases sensitivity of JEG-3 and JAR to 5-FU.

## Insulin sensitizes CC cells to 5-FU *in vivo*

**Figure 2.**
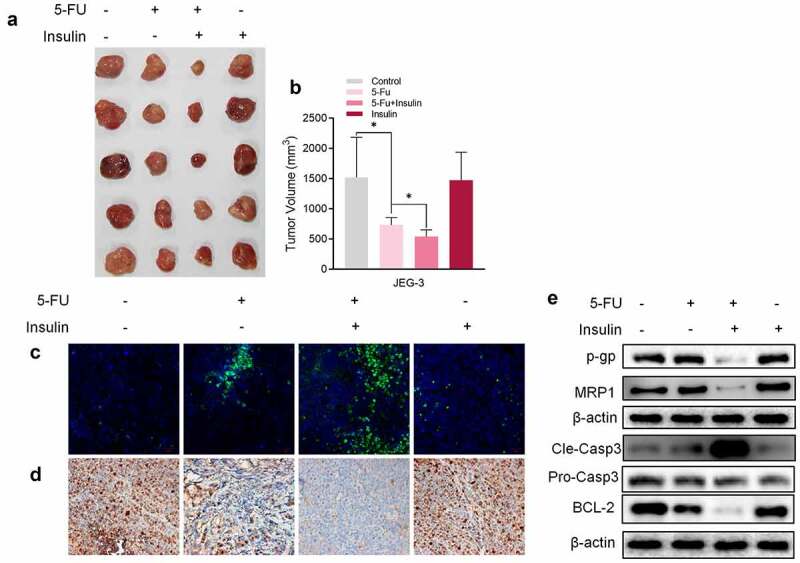
Insulin sensitizes choriocarcinoma cells to 5-FU *in vivo*. (a) Representative images of xenograft tumors isolated from nude mice in the different groups. (b) Tumor sizes in different groups. (c) TUNEL assay of xenograft tumors tissue. (d) Immunohistochemistry of Ki-67 in xenograft tumors tissue. (e) Western blot analysis showing protein expression levels of p-gp, MRP1, cleaved caspase-3, pro caspase-3, and BCL-2 in xenograft tumor tissue. β-Actin was used as the internal reference. Data are expressed as mean ± SEM (*n* = 5; *p < 0.05)

To explore the association between insulin and 5-FU resistance *in vivo,*CC cells, JAR was implanted into nude mice by subcutaneous injection. After implantation of CC cells, mice were administered with 5-FU (20 mg/kg) or/and insulin every two days. A single dose of insulin had no effect on the volume of mice tumors, whereas co-administration of insulin with 5-FU showed significantly higher effectiveness of tumor inhibition compared with administration of 5-FU alone (Figure 2a, b). In addition, analysis of expression levels of TUNEL (Figure 2c), Ki-67 (Figure 2d), and caspase-3 (Figure 2e) showed that co-administration of insulin with 5-FU significantly promoted tumor apoptosis and inhibited tumor proliferation compared with administration of 5-FU alone. Furthermore, western blot results showed that insulin suppressed chemoresistance-related proteins P-GP and MRP1, which was consistent with *in vitro* results (Figure 2e). These findings show that insulin sensitizes CC cells to 5-FU in xenograft tumor mice.

## Insulin sensitizes CC cells to 5-FU by modulating phosphorylation of survivin

**Figure 3.**
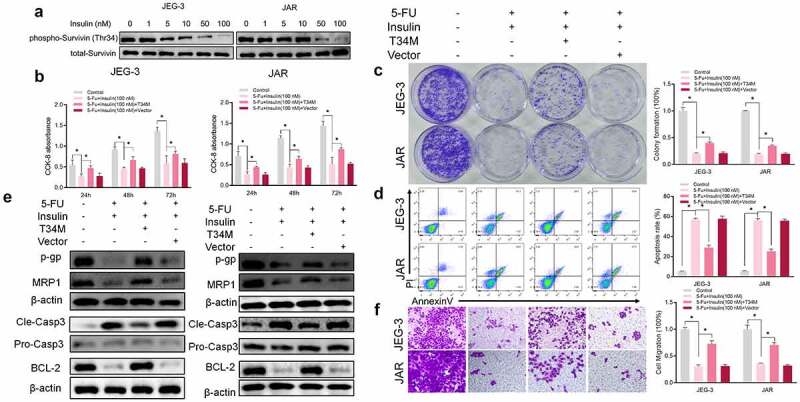
Insulin sensitizes choriocarcinoma cells to 5-FU by modulating phosphorylation of Survivin. (a) Western blot analysis showing protein expression of level of survivin and phosphorylation (Thr34) of survivin in JEG-3 and JARS cells. (b CCK-8 assays were performed to evaluate cell proliferation at different times. (c) Colony formation assay showing proliferation in JEG-3 and JARS cells after transfection with survivin Thr34 phosphomimetic mutant vector (T34M) and culturing in 5-FU or insulin for 48 h. (d) Quantification and analysis of apoptosis rates in JEG-3 and JARS cells after transfection with T34M and culturing in 5-FU or insulin for 48 h using flow cytometry. (e) Western blot analysis showing protein expression levels of p-gp, MRP1, cleaved caspase-3 (Cle-Casp3), pro caspase-3 (Pro-Casp3), and BCL-2 in JEG-3 and JARS cells. β-Actin was used as the internal reference. (f) Cells migration rates detection using Transwell assay. Data are expressed as mean ± SEM (*n* = 3; *p < 0.05)

Survivin is a member of the inhibitor of apoptosis protein (IAP) family and is the most potent inhibitor of apoptosis reported so far [19,20]. Mesri et al [21] reported increased mitochondrial release of cytochrome *C* and caspase-3 activity and increased apoptosis in tumor cells, after mutation of threonine 34 (Thr34) phosphorylation site of survivin in breast, cervical, prostate, lung, and colorectal cancer cell lines. The findings of the current study show that insulin suppressed phosphorylation of survivin at residue Thr34 in a dose-dependent manner in CC cells (Figure 3a). To further validate the role of Thr34 on the role of insulin in increasing sensitivity to 5-FU in CC, we established CC cells that stably expressed survivin–Thr34–mutant (phosphomimetic mutant, T34M) and survivin–vector (Vector). CCK-8 assay (Figure 3b) and colony formation (Figure 3c) showed that T34M reversed chemotherapy sensitivity of insulin to 5-FU in JEG-3 and JAR cells. Annexin V staining followed by flow cytometry showed decreased apoptosis in T34M compared with the wild type (Figure 3d). A significant increase in resistance family protein expression was observed after T34M transfection (Figure 3e). Moreover, cell migration showed a similar pattern and cell migration rate was significantly higher in T34M compared with the wild type (figure 3f). These findings indicate that sensitization of insulin to 5-FU is through modulation of phosphorylation of survivin at Thr34.

Insulin reversed chemoresistance to 5-FU in CC cells. Insulin combined with 5-FU suppressed CC tumor progression in xenograft mice. Insulin inhibited phosphorylation of survivin at residue threonine 34 in CC cells.

## Discussion

In normal pregnancy, trophoblast cells exhibit invasiveness similar to that presented by malignant tumor cells, but this invasive ability is regulated by the body. However, when the regulation is disturbed and trophoblast cells become highly invasive, the patient may develop CC. CC is a highly metastatic trophoblastic tumor. Incidence of CC is 1 in every 24,096 pregnancies [22], and it is characterized by risk of brain and lung metastases. Although efficacy of chemotherapy for CC has been extensively explored, 10–20% of patients with CC are resistant to treatment or present with high rates of recurrence due to a single metastatic lesion. Therefore, understanding potential recurrence and metastatic mechanisms of CC is important for improving the survival rates of CC patients.

Most type 2 diabetes mellitus (T2DM) patients need exogenous insulin therapy during clinical treatment; therefore, their serum insulin levels are higher compared with non-diabetic people [23,24]. Insulin is a growth factor that plays an important role in cell proliferation, differentiation, and apoptosis. In addition, insulin is implicated in tumorigenesis and tumor development [25]. Recent epidemiological studies report that people with T2DM are at a higher risk of developing neoplastic diseases. For instance, the relative risk ratio (RR) of colon cancer is 1.4, breast cancer RR is 1.1, pancreatic cancer RR is 2.2, and liver cancer RR is 2.1 [26–29]. However, risk of developing CC in patients with T2DM compared with other tumors has not been explored. Previous studies have reported correlation of insulin with tumor resistance. Interestingly, insulin resistance to 5-FU has both positive and negative effects in different tumors. Insulin increases cytotoxicity of 5-FU in breast cancer cells through activation of apoptotic and autophagic pathways [30]. In addition, it has been reported that insulin enhances anticancer functions of 5-FU in esophageal and colonic cancer cells [31]. On the contrary, insulin was found to confer anti-apoptotic effect on gastric cancer cell lines treated with 5-FU [32]. In this study, insulin increased cytotoxic effects of CC cells to 5-FU through suppression of resistance-associated protein and promotion of apoptosis. This effect offer an advantage for patients with type 2 diabetes that are under CC medication. Studies have also found that extremely high insulin levels in humans trigger a series of phosphorylation events that lead to the activation of multiple pathways, including the PI3K pathway, and ultimately to a highly proliferative and invasive cancer phenotype. High doses of insulin are known to activate IGF1R-related signaling and affect the propensity to invade and metastasize [33,34]. However, the use of insulin alone in our study did not have an effect on the progression of CC cells. These apparently conflicting results may be related to the fact that the concentration of insulin used in the *in vitro* experiments did not correspond to the concentration in the clinical setting. As a future study, we will study in depth the effects of insulin targeting pathways such as PI3K in CC cells.

Studies report that sensitivity of tumor cells to chemotherapy is related to apoptosis, and chemical drugs induce apoptosis mainly by inhibiting growth of tumor cells. Survivin is a tumor-specific apoptosis inhibitor gene and a direct inhibitor of caspase-3 and caspase-7, thus it blocks apoptotic process [35]. It has been reported that survivin is expressed in human villous trophoblast, villous mesenchymal cells, and villous carcinoma cell lines [36]. High expression level of survivin has been reported in CC cell line. Survivin binds to the cell cycle regulator CDK4, resulting in CDK2/cyclin-E activation and ribosomal phosphorylation. Phosphorylation initiates entry of cells into the cell cycle, accelerates G1/S phase transition, releases P21 from the survivin–CDK4 complex, binds to mitochondrial pro-caspase-3, and inhibits caspase-3 activity, thus preventing mitochondrial release of cytochrome *C*, which ultimately inhibits apoptosis [37,38]. Our data of current research showed that co-administration of insulin with 5-FU inhibited caspase-3 activity. Therefore, the inhibitory effect of insulin on 5-FU resistance may be related to regulation of caspase-3 by CDK4. However, this claim should be explored in further studies. In addition to 5-FU, methotrexate, vincristine, cyclophosphamide, and adriamycin are used in clinical chemotherapy treatment of CC [2]. Therefore, further studies should explore the effect of insulin on these chemotherapeutic agents to fully address limitation of chemoresistance in CC.

## Conclusion

In conclusion, the findings of this study show that insulin reverses chemoresistance of CC cells to 5-FU by inhibiting phosphorylation of survivin. A novel therapy from combination of insulin with chemotherapeutic drug 5-FU will potentially improve survival of CC patients.
